# Membranoproliferative glomerulonephritis in a patient with lysinuric protein intolerance: lesson for the clinical nephrologist

**DOI:** 10.1007/s40620-024-02018-2

**Published:** 2024-07-17

**Authors:** Demet Baltu, Oğuzhan Serin, Tekin Aksu, Hayriye Hızarcıoğlu Gülşen, Diclehan Orhan, Yılmaz Yıldız, Didem Yücel Yılmaz, Doğuş Vurallı, Yelda Bilginer, Bora Gülhan, Ali Düzova

**Affiliations:** 1 Division of Pediatric Nephrology, Hacettepe University Faculty of Medicine, Sihhiye, 06100 Ankara, Turkey; 2 Department of Pediatrics, Hacettepe University Faculty of Medicine, Ankara, Turkey; 3 Division of Pediatric Hematology, Hacettepe University Faculty of Medicine, Ankara, Turkey; 4 Division of Pediatric Gastroenterology, Hepatology and Nutrition, Hacettepe University Faculty of Medicine, Ankara, Turkey; 5 Department of Pathology, Hacettepe University Faculty of Medicine, Ankara, Turkey; 6 Division of Pediatric Metabolism, Hacettepe University Faculty of Medicine, Ankara, Turkey; 7 Division of Pediatric Metabolism, Hacettepe University Institute of Child Health, Ankara, Turkey; 8 Division of Pediatric Endocrinology, Hacettepe University Faculty of Medicine, Ankara, Turkey; 9 Division of Pediatric Rheumatology, Hacettepe University Faculty of Medicine, Ankara, Turkey

**Keywords:** Lysinuric protein intolerance, IC-MPGN, Inborn error of metabolism, Nephrotic syndrome, Glomerulonephritis

## The case

An 11-year-old girl, the second child of healthy consanguineous parents, was admitted to our hospital for evaluation of recurrent fever and hepatosplenomegaly lasting since about one year. Her past medical history revealed left femur fracture at 8 months of age and a left tibia fracture at 3 years of age with minor trauma. On initial physical examination her weight was 26 kg (– 1.94 SDS), height was 143 cm (– 0.01 SDS), body mass index was 12.71 (– 3.05 SDS), and hepatosplenomegaly was noted. Laboratory investigations revealed pancytopenia (hemoglobin 9.4 g/dL, white blood cells 3400/µL, platelets 61,000/µL), high ferritin [9687 ng/mL (11–307)], and lactate dehydrogenase [2517 U/L (110–25)] levels. Bone marrow aspirate showed hemophagocytosis. Dexamethasone, cyclosporine, and intravenous immunoglobulins were initiated following the diagnosis of hemophagocytic lymphohistiocytosis (HLH). The amino acid profile was characteristic for lysinuric protein intolerance (LPI), with strikingly elevated urinary lysine (19,090 µmol/g cre, N: < 190), arginine (4097 µmol/g cre, N: < 120), and ornithine (3216 µmol/g cre, N: < 150), and moderately elevated plasma glycine (571 µmol/L, N: < 300) and alanine (846 µmol/L, N: < 500). Polymerase chain reaction failed to amplify exons 5 through 10 of the *SLC7A7* gene (RefSeq NM_003982), suggesting homozygous rearrangement of this region, previously reported by others to cause lysinuric protein intolerance [1]. As treatment of lysinuric protein intolerance, L-citrulline, L-carnitine, and sodium benzoate were initiated, along with a protein-restricted diet.

**Fig. 1:**
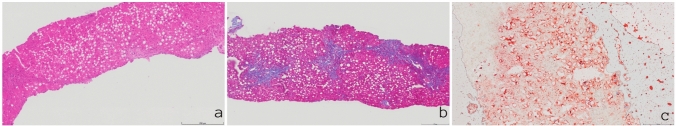
**a** Macrovesicular steatosis in liver biopsy (Hematoxylin and Eosin, scale bar = 250 μm) **b** Marked fibrosis and porto-portal-porto-central bridging fibrosis (Masson Trichrome, scale bar = 200 μm) **c** Lipid deposition in hepatocytes in frozen sections (Oil-red-O, scale bar = 200 μm)

**Fig. 2:**
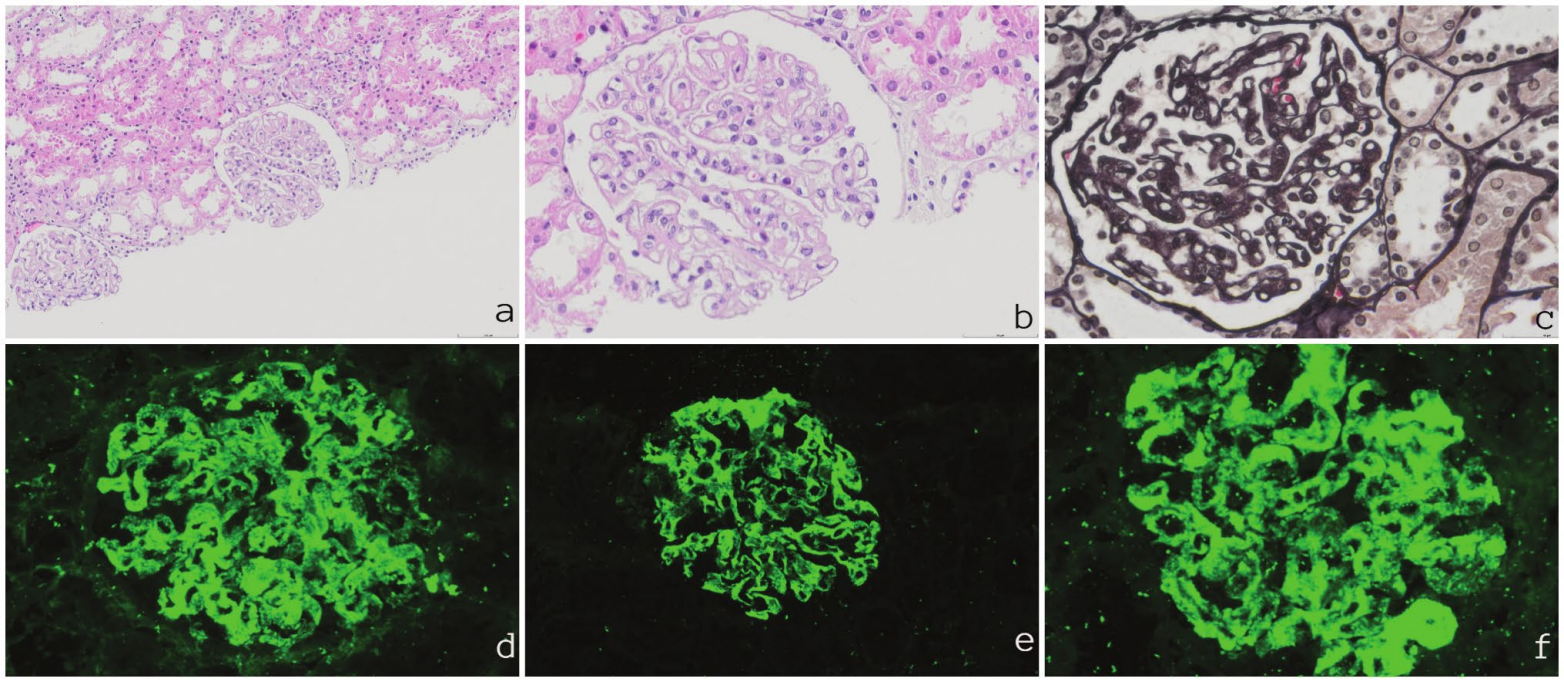
**a** Mesangial hypercellularity with increase in mesangial matrix (Hematoxylin and Eosin, scale bar = 100 μm) **b **Irregular thickening of glomerular basement membranes (Hematoxylin and Eosin, scale bar = 50 μm) **c** Glomerular basement membrane double contours (Jones Methenamine Silver, scale bar = 50 μm) Irregular granular capillary loop and mesangial immunofluorescence staining with C3 (**d** scale bar = 50 μm), IgG (**e** scale bar = 50 μm), IgM (**f** scale bar = 50 μm)

Her clinical status remained stable during the following 2 years. Then, at the age of 13 years, she was admitted to the hospital with complaints of abdominal pain and distension. Physical examination revealed pallor, ascites and hepatosplenomegaly. A liver biopsy was performed and the pathological findings were compatible with macrovesicular steatosis (Fig. 1). During hospitalization, she developed nephrotic syndrome (hypoalbuminemia, albuminuria 6804 mg/day). Tubular proteinuria (beta-2 microglobulin level 14,178 ng/mL (0–300)) was also observed. Proximal tubular acidosis, hypercalciuria, and phosphaturia were not observed. Complement 3 (C3) and complement 4 (C4) levels were decreased (48 mg/dl (79–152), < 1.67 mg/dl (16–38), respectively). Anti-nuclear antibody (ANA) was 1/160 and anti-double-stranded DNA was negative. Serum creatinine and urine output were normal. Kidney biopsy showed immune complex membranoproliferative glomerulonephritis (IC-MPGN) with light microscopy and immunofluorescence findings (Fig. 2). Electron microscopy could not be performed due to lack of availability in our center.

There was no evidence of infectious disease, systemic immune disease, or malignancy, and C3 nephritic factor was negative and a search for complement gene mutations was not performed. She was treated with pulse methylprednisolone (15 mg/kg/day, 3 days), and then treatment continued with oral prednisolone (0.5 mg/kg/day). Angiotensin-converting enzyme inhibitor (ACEi) was started. Proteinuria decreased to 22 mg/ m^2^/h in the first month and complete remission was achieved in the third month of treatment. Prednisolone was tapered and stopped within 18 months. She has been in remission with only ACEi for the last seventeen months. Other laboratory parameters are presented in Table 1.

**Table 1:** Laboratory findings at IC-MPGN diagnosis and at last visit

	Diagnosis	Last visit	Reference range
Complete blood count			
Hemoglobin, g/dl	9.70	9.20	11.7–15.5
White blood cells, 10^3^/µL	7.80	7.00	4.1–11.2
Platelets, 10^3^/µL	171	182	159–388
Biochemistry			
Sodium, mEq/L	134	138	136–146
Potassium, mEq/L	4.18	4.2	3.4–4.7
Calcium, mg/dl	9.36	9.49	8–8-10.8
Phosphorus, mg/dl	2.76	4.52	3.2–5.7
Creatinine, mg/dl	0.23	0.34	0.26–0.77
Blood urine nitrogen, mg/dl	13.50	7.4	5–18
Uric acid, mg/dl	3.07	3.2	2.6–6
ALT, U/L	58	21	< 39
AST, U/L	192	77	< 51
GGT, U/L	289	75	4–22
Total bilirubin, mg/dl	0.48	0.38	0.3–1.2
Albumin, g/dl	2.76	3.52	3.5–5.2
Urine tests			
pH	8	5.5	
Density	1015	1009	
Protein	+ + +	Negative	
Glucose	Negative	Negative	
Leukocytes	5	1	
Erythrocytes	3	1	
Beta-2 microglobulin, ng/mL	14,178	NA	0–300
Protein (24-h urine), mg/day	12,058.2	191.4	< 80
Albumin (24-h urine), mg/day	6804	11.11	< 30
Other laboratory tests			
Complement 3, mg/dl	48	NA	79–152
Complement 4, mg/dl	< 1.67	NA	16–38
ANA	1/160		
Anti-ds DNA	Negative		

*ALT* alanine aminotransaminase, *ANA* Anti-nuclear antibody, *anti-ds DNA* anti-double-stranded DNA antibody, *AST* aspartate aminotransferase, *ESR* erythrocyte sedimentation rate, *GGT* gamma glutamyl transferase, *IC-MPGN* immune complex membranoproliferative glomerulonephritis, *MCHC* mean corpuscular hemoglobin concentration, *MCV* mean corpuscular volume, *NA* not available

In the fourth and fifth month after the kidney biopsy, the patient developed cytopenia with high ferritin, LDH, and low fibrinogen levels. Bone marrow evaluation revealed recurrence of hemophagocytosis. Intravenous immunoglobulin treatment was initiated. After these two attacks, hemophagocytic lymphohistiocytosis did not develop in the follow-up. Hepatosplenomegaly was still present on physical examination. The bone fractures did not recur, under treatment with vitamin D and calcium, but she developed scoliosis. Estradiol treatment was started upon detection of hypogonadotropic hypogonadism, which was thought to be secondary to chronic disease. Esophagogastroduodenoscopy showed stage 2–3 varicose veins in the lower 1/3 of the esophagus and propranolol treatment was started. Currently, her clinical status is stable under multidisciplinary care.

## Lessons for the clinical nephrologist

Herein we report a patient with a diagnosis of lysinuric protein intolerance who developed nephrotic syndrome accompanied by low C3 and C4. Immune complex-MPGN, lupus nephritis (LN), and C3 glomerulopathy were considered in the differential diagnosis before biopsy. The patient had no clinical findings compatible with systemic lupus erythematosus, except ANA positivity, and kidney biopsy was not suggestive of LN. The C3NeF was negative and there were deposits of co-dominant immunoglobulins/immune complex as well as C3 accumulation which excluded C3 glomerulopathy. Based on the laboratory and kidney biopsy findings, we diagnosed IC-MPGN and considered it to be related to lysinuric protein intolerance. Thereafter, she achieved remission with steroids and ACEi.

Lysinuric protein intolerance is a rare autosomal recessive metabolic disease caused by mutations in the *SLC7A7* gene, disrupting the function of the cationic (y + L) amino acid transporter-1 (y + LAT-1), a transport protein of dibasic amino acids (lysine, arginine, and ornithine). Defective transport of these amino acids in the intestine and kidney leads to amino acid imbalances, disturbing multiple pathways, including protein synthesis and the urea cycle. A transport defect in non-polarized cells (lymphocytes or macrophages) is also likely implicated in the pathogenesis [2].

Lysinuric protein intolerance is a disease with multisystem involvement. Growth delay, recurrent fractures, hepatosplenomegaly, cirrhosis, diarrhea, hemophagocytic lymphohistiocytosis, pulmonary alveolar proteinosis (PAP), vascular lesions (cerebral infarction), pancreatitis are other clinical findings [2]. Kidney involvement may also be observed. Generally, it begins with mild proteinuria and hematuria and can progress to kidney failure. Tanner et al. described 39 patients with lysinuric protein intolerance with a mean age of 30.5 years (range, 1–62 years) and observed proteinuria in 74% and hematuria in 38%. Kidney failure developed in 4 patients, 2 of whom were < 25 years old [3]. Although kidney involvement is often tubulo-interstitial, glomerular involvement has been reported [4, 5].

The glomerular involvement in lysinuric protein intolerance ranges from lupus-like lesions to amyloidosis, according to published data [6]. Recent data from a series of five patients who underwent kidney biopsy between 1986 and 2015 mainly describe tubulointerstitial nephritis and nephrocalcinosis but one of the five patients showed a MPGN pattern [4]. Although immune complex glomerulonephritis (IC-GN) in lysinuric protein intolerance has been reported, in most cases this diagnosis was made on the basis of autopsy or kidney biopsy findings performed shortly before death. In a few cases, immunosuppressive treatment was attempted, but kidney response could not be assessed because the patients died shortly afterwards of various causes, including pulmonary alveolar proteinosis, hemophagocytic lymphohistiocytosis, septic shock, and multiorgan failure. To the best of our knowledge, there is only one patient whose response to immunosuppressive treatment for IC-GN has been documented in the literature. As in our patient, this patient presented with nephrotic syndrome and was followed for approximately 4 years. Initially, remission was achieved with steroid and mycophenolate mofetil (MMF) treatment, but later a relapse occurred after the steroid treatment was stopped, and therefore, steroid treatment was resumed. Since then, partial remission was achieved with persistent proteinuria (approximately 1 g/d) on steroid and MMF treatment [4, 5]. Our patient developed nephrotic syndrome and steroid treatment was initiated after the kidney biopsy. By the third month, the patient was in complete remission. Steroid treatment was tapered and discontinued after 18 months and she was followed for 3 years after the kidney biopsy and has been in remission without treatment for the last 17 months. This result supports that IC-MPGN, which is very rarely seen in patients with lysinuric protein intolerance, can benefit from immunosuppressive treatment.

Immune dysregulation is a well-known complication in lysinuric protein intolerance that has been associated with hemophagocytic lymphohistiocytosis, autoimmune diseases, and pulmonary alveolar proteinosis. The underlying mechanism is still obscure. Involvement of the mononuclear phagocytic system appears to be the main factor. One of the culprits thought to be involved in the pathophysiology is the nitric oxide (NO) pathway. Nitrate oxide has significant effects on the immune system, cytokine production, and cyclooxygenase activation. Because of defective efflux through the cationic amino acid transporter, arginine accumulates in the monocytes/macrophages of lysinuric protein intolerance patients, causing increased NO synthesis. Manucci et al. [7] reported increased plasma NO levels in patients with lysinuric protein intolerance. Therefore, it was hypothesized that increased NO is a major initial trigger of activation of the immune system and immune abnormalities in lysinuric protein intolerance patients. On the other hand, Rotoli et al. [8] showed increased inflammation in *SLC7A7*-silenced monocytes, that was independent of intracellular arginine concentration.

In conclusion, although tubular dysfunction, nephrocalcinosis, and chronic tubulointerstitial nephritis are the most common forms of kidney involvement in lysinuric protein intolerance, glomerular involvement with immune-mediated glomerulonephritis may also occur, and immunosuppressive therapy may be indicated.

## Data Availability

All data have been presented in the manuscript.
